# Therapeutic Potential of Pistachio Green Hull Extract in Treating Parkinson's Disease: A Comprehensive In Vivo and In Vitro Investigation

**DOI:** 10.1002/fsn3.70204

**Published:** 2025-05-16

**Authors:** Maedeh Tavakoli, Negin Mohammadi, Mohsen Barzegar, Neda Valian, Narges Hosseinmardi, Mohammad Ali Sahari, Sabereh Saremi

**Affiliations:** ^1^ Department of Food Science and Technology Tarbiat Modares University Tehran Iran; ^2^ Neuroscience Research Center Shahid Beheshti University of Medical Sciences Tehran Iran; ^3^ Department of Physiology, Medical School Shahid Beheshti University of Medical Sciences Tehran Iran; ^4^ Department of Biochemistry Tarbiat Modares University Tehran Iran

**Keywords:** 6‐OHDA, α‐synuclein, antioxidant, behavioral tests, Parkinson's disease, pistachio green hull extract

## Abstract

Pistachio green hull extract (PGHE), an agro‐industrial by‐product, is rich in phenolic compounds with significant inherent antioxidant and anti‐inflammatory activities. In this study, the neuroprotective effects of PGHE against Parkinson's disease (PD), a progressive neurodegenerative disorder, were evaluated both in vivo and in vitro. The lethal dose (LD_50_) of PGHE was greater than 5000 mg/kg, indicating its safety and nontoxicity. Rats were orally treated with PGHE (800 mg/kg/day) 24 h after injection of 6‐OHDA (20 μg/rat in right MFB) for 14 days. Motor function was evaluated on Days 7 and 15 after 6‐OHDA administration using the cylinder, narrow beam, pole, rotarod, and apomorphine‐induced rotations tests. PGHE significantly improved motor impairments induced by 6‐OHDA. An in vitro study indicated that increasing concentrations of PGHE up to 250 μg/mL resulted in a reduction in the aggregation of α‐synuclein characterized by thioflavin T fluorescence and Congo red assays. Transmission electron microscopy images further confirmed a decrease in α‐synuclein aggregation in the presence of PGHE. In conclusion, these findings indicated that PGHE improved motor deficits in a rat model of PD and decreased α‐synuclein aggregation in vitro, suggesting that it can be considered a novel dietary supplement for PD therapy. However, more studies are needed to clarify the underlying mechanisms of PGHE's effect on PD pathogenesis and its potential applications in developing functional foods or nutraceuticals.

## Graphical abstract

A new natural extract (PGH) was introduced for the treatment of Parkinson disease (PD). PGHE treatment of PD in a rat model led to better behavioral results. PGHE has a neuroprotective effect in the 6‐OHDA‐induced model of PD, and at 250 μg/ml had the highest inhibitory effect on α‐synuclein fibrillation.

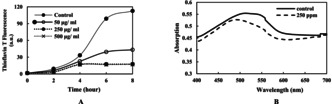

**Abbreviations**
6‐OHDA: 6‐Hydroxydopamine hydrochloride
α‐syn: α‐synuclein
ROS: reactive oxygen species
PD: Parkinson`s disease
PGH: pistachio green hull
BW: body weight
PGHE: pistachio green hull extract
IPTG: isopropyl‐β‐D‐thiogalactoside
SNpc: substantia nigra pars compacta
LD_50_
: lethal dose
ThT: thioflavin T
MFB: medial forebrain bundle

## 1 Introduction

Parkinson's disease (PD) is the second most prevalent neurodegenerative disease after Alzheimer's and affects approximately 1% of the adult population over 60 years of age (Ju et al. 2010; Perez‐Pardo et al. 2017). The characteristic pathological hallmarks of PD are the loss of the dopaminergic neurons in the substantia nigra pars compacta (SNpc) of the midbrain and the formation of Lewy bodies containing α‐synuclein (α‐syn) aggregates in the cytoplasm of surviving neurons, leading to characteristic motor disorders (Feng et al. 2019; Hemmati‐Dinarvand et al. 2019; Perez‐Pardo et al. 2017). Consequently, PD appears through behavioral characteristics such as postural instability, bradykinesia, tremors, and rigidity. Additionally, it affects non‐motor functions as well (Beserra‐Filho et al. 2019; Li et al. 2021). However, the underlying mechanisms responsible for the dopaminergic neurons' selective degeneration and the PD cause remain unknown. Reactive oxygen species (ROS) overproduction is thought to play a key role in oxidative damage, the unusual processing of proteins such as the agglomeration of α‐syn, and finally the apoptosis of dopaminergic neurons. Consequently, a reduction in dopamine production ability occurs (Chang et al. 2019; Pignolo et al. 2022). Intense metabolic levels along with the high capacity of oxidizable species involving iron and polyunsaturated fatty acids in the dopaminergic neurons lead to oxidative stress damage of the dopaminergic neurons (Hemmati‐Dinarvand et al. 2019). Besides, α‐syn is one of the proteins responsible for generating reactive species within mitochondria. α‐syn, through binding to the external membrane of mitochondria, causes mitochondrial dysfunction. Therefore, reactive species lead to oxidative stress and stimulate the release of inflammatory cytokines from microglia and astrocytes, ultimately resulting in neuronal degeneration (Feng et al. 2019). Meanwhile, deficiency of the antioxidant constituents in daily diets can increase ROS levels leading to PD onset and development.

Unfortunately, Parkinson's disease is not curable by conventional medications. These medications can only reduce some symptoms. For example, Levodopa (L‐dopa), as an effective drug for the first motor symptoms of PD, can reduce stiffness and bradykinesia, but other symptoms such as balance problems may become worse. Furthermore, patients need higher L‐dopa doses over time, leading to an increase in side effects like dyskinesias and motor disorders. Therefore, innovative therapeutic approaches need to prevent PD's effects on the brain, such as neurodegeneration and α‐syn aggregation.

Therefore, the utilization of complementary therapies and phytonutrients is recommended for PD treatments (Mittal et al. 2023). For this reason, components with anti‐inflammatory and antioxidant properties can be used to restrict the oxidative stress damage pathway (Pignolo et al. 2022).

Antioxidants such as flavonoids, vitamins, and polyphenols have an inherent ability to protect neurons from oxidation by protecting or repairing cellular constituents (Mittal et al. 2023). For these reasons, many researchers became interested in this field, and several studies investigated the neuroprotective effects of natural antioxidants, especially plant‐derived phenolic compounds such as ellagic acid, myricetin, tyrosol, and curcumin for the treatment of PD and to reduce its symptoms (Baluchnejadmojarad et al. 2017; García‐Moreno et al. 2019; Huang et al. 2018; Jin et al. 2022). Thus, according to previous studies, phenolic compounds can have significant positive effects on PD.

Reports showed that processing industries produce significant amounts of plant‐based by‐products that are rich sources of polyphenolic compounds. Thus, these agro‐industrial by‐products can be used as antioxidants to promote beneficial health effects (Lalegani et al. 2018).

In the world, Iran is the major producer and exporter of pistachios. Pistachio green hull (PGH) is the by‐product of industrial pistachio production and a cost‐effective source of phenolic compounds such as gallic acid, phloroglucinol, naringin, and vanillic acid, which have considerable antioxidant activities (Ghandahari Yazdi et al. 2019). Already, several studies have reported the remarkable functional properties and health‐promoting benefits of pistachio green hull extract (PGHE). These features include acting as antioxidant and antimicrobial agents in foodstuffs, etc. (Goli et al. 2005; Rafiee et al. 2018; Rajaei et al. 2010; Sadeghinejad et al. 2019; Sarteshnizi et al. 2019), anti‐inflammatory (Grace et al. 2016), anti‐mutagenicity (Rajaei et al. 2010), anti‐diabetic (Lalegani et al. 2018; Sarteshnizi et al. 2021), cellular protection of lymphocytes and erythrocytes and prevention of protein degradation (Barreca et al. 2016), and hypertension prevention (Sila et al. 2014). Thus, these studies introduce PGH as a cheap and remarkable source of health‐promoting compounds that have the potential to be used in food supplements and medicines. Furthermore, gallic acid is the main constituent of PGHE, and its neuroprotective effect by antioxidant defense enhancement and attenuating neuroinflammation has previously been reported (Liu et al. 2020; Mansouri et al. 2013). Therefore, we assume that PGHE may have anti‐PD properties due to its high antioxidant activity by restricting the oxidative stress damage of the dopaminergic neurons.

In this study, we aimed to evaluate the effects of PGHE on Parkinson's disease. For this purpose, (i) PGHE was used and examined in the Wistar rat model of PD using behavioral tests to assess its effect and (ii) the possible pathway affecting Parkinson's disease was examined by evaluating the PGHE effect on α‐synuclein aggregation in vitro. This served as an indicator of intracellular toxicity development in the brain tissue.

## 2 Material and Methods

### 2.1 Materials

PGH (*Ahmad Aghaei* variety) was obtained from the Kerman Agricultural Research Center of Iran. 6‐hydroxy dopamine hydrochloride (6‐OHDA) (Cat# H4381), Congo red (Cat# 234610), and Thioflavin T (Cat# 596200) were purchased from Sigma‐Aldrich (USA). Apomorphine hydrochloride hemihydrate was from Santa Cruz Biotechnology Co. (Cat# sc‐253341, USA). Desipramine‐HCl was purchased from Pars Daroo Co. (Tehran, Iran). Isopropyl‐β‐D‐thiogalactoside (IPTG) was obtained from the Molekula group (Cat# 21689530, France). All other chemicals purchased from Merck Chemical Co. (Darmstadt, Germany) were analytical grade.

### 2.2 PGHE Preparation

PGH powder and distilled water (1:15 ratio) were blended and agitated (8 h, at 25°C). Subsequently, the mixture was centrifuged (10 min, 3000 g), and the resulting supernatant was filtered using Whatman No. 42. The filtered aqueous extract was spray‐dried, and the obtained PGHE powder was preserved in a sealed container at −20°C until in vivo and in vitro investigations (Rajaei et al. 2010). The PGHE powder was dissolved in distilled water for animal treatments and in vitro investigations.

### 2.3 LC/MS Determination of PGHE Phenolic Compounds

The analysis of PGHE phenolic compounds was performed using an LC/MS instrument (an Agilent 6150 single quad mass spectra, equipped with an agilent1260 binary pump, degasser, column heater at 40°C, and 1367C autosampler). A waters XBridge C18 column measuring 150 × 4.6 mm with a particle size of 5 μm was employed for chromatographic separation. Solvents A and B consisted of H_2_O + 0.1% (v/v) formic acid and acetonitrile +0.1% (v/v) formic acid, respectively. The gradient conditions were utilized as follows: 0–3 min, 0% B; 3–9 min, 3% B; 9–24 min, 12% B; 24–30 min, 20% B; 30–33 min, 20% B; 33–43 min, 30% B; 43–63 min, 40% B; 63–67 min, 100% B; 67–72 min, 100% B. Subsequently, the system was balanced for 7 min, resulting in an overall run time of 72 min. The flow was at a rate of 1.0 mL/min, with an injection volume of 5 μL. UV–Vis spectra of PGHE phenolic compounds were recorded at 260 nm. The identification of these compounds was done by comparing their retention times, UV–Vis spectra, scan mass spectra (50–1350 amu), and MS fragmentation patterns with their corresponding standards analyzed under the same conditions, along with previous literature reports (Barreca et al. 2016).

### 2.4 Animals

Male Wistar rats were from the breeding colony of the Neuroscience Research Center, Shahid Beheshti University of Medical Sciences (Tehran, Iran). All animals were adapted to laboratory conditions for 2 weeks. The schedule of the animal room was a 12:12 h light/dark cycle at 23°C ± 2°C. The rats had open access to water and food.

### 2.5 Acute Oral Toxicity of PGHE

According to Lork's method (Chinedu et al. 2013), nine animals were divided into three groups of three animals each in Phase 1. Each group of animals was orally administered various single doses of PGHE (10, 100, and 1000 mg/kg). The animals were monitored for 24 h for mortality and behavioral observations. After that, three animals were divided into three groups of one animal each. The rats were orally administered higher doses of PGHE at 1600, 2900, and 5000 mg/kg. They were followed for 24 h for mortality and signs of behavioral change. Moreover, all the animals in the two mentioned phases were observed for 14 days.

### 2.6 Surgical Procedures and Experimental Groups

To induce the PD model, animals received 6‐OHDA neurotoxin into the right medial forebrain bundle (MFB). 6‐OHDA is usually used to induce an animal model of PD in experimental studies (Chia et al. 2020; Schober 2004). Desipramine (25 mg/kg, i.p.) was administered 30 min before the surgery to prevent 6‐OHDA uptake into noradrenergic terminals. Afterward, the rats were anesthetized by ketamine/xylazine (100/10 mg/kg, i.p.) and were fixed in a stereotaxic surgery apparatus. 6‐OHDA was dissolved in normal saline 0.9% containing 0.02% ascorbic acid and injected in the right MFB (20 g/5 l) at the following coordinates according to the brain atlas of Paxinos and Watson (2006): anterior–posterior (AP) = 4.3 mm, mediolateral (ML) = 1.8 mm dorsoventral (DV) = 8.2 mm. In the sham group the same volume of the vehicle (5 l) was injected into the right MFB.

The treatments started after 24 h of the surgery and continued for 14 days (Figure S1). The animals were orally treated with PGHE (800 mg/kg BW/day). Therefore, adult male Wistar rats (220–270 g) were randomly divided into four groups (*n* = 7‐9/group): (1) sham (vehicle in MFB, treated by distilled water, p.o.), (2) 6‐OHDA (6‐OHDA in MFB, treated by distilled water, p.o.), (3) 6‐OHDA + PGHE 800 (6‐OHDA in MFB, treated by 800 mg/kg BW/day PGHE, p.o.), and (4) PGHE 800 (vehicle in MFB, treated by 800 mg/kg BW/day PGHE, p.o.).

### 2.7 Behavioral Tests

Behavioral experiments were performed 7 and 15 days after the surgery. Before the surgery, all animals were trained for two consecutive days for narrow beam, pole, and rotarod tests.

#### 2.7.1 Cylinder Test

A cylinder test, namely the forelimb asymmetry test, was done to determine the forelimb use preferences. The animals were placed into the clear plexiglass (diameter 20 cm and height 35 cm). After that, the number of ipsilateral and contralateral forelimbs touching the wall of the cylinder in 5 min was recorded and then counted. Data were presented as the use of contralateral forelimb percentage (Soner et al. 2021).

#### 2.7.2 Narrow Beam Test

To evaluate the balance and motor coordination of animals, we used the narrow beam test. A wooden beam apparatus measuring 100 cm in length, width of 4 cm, and height of 3 cm was used for this assessment. The beam was placed 80 cm above the ground. From the start of the beam, a 20 cm line was drawn, and a home cage was located on the other end of the beam. In days of training and testing, each rat was entirely placed within the 20 cm starting zone facing its home cage. A stopwatch was immediately started upon the release of the rat. The time to pass the start zone showed the latency to begin the task (time to start walking). The total time on the beam was then recorded when all 4 feet were placed upon the home cage. The time to start walking and total time were recorded in 3 trials for each rat; the average of 3 trials was considered as the final score. The time cut‐off for each rat to do this task was 120 s (Allbutt and Henderson 2007).

#### 2.7.3 Pole Test

To perform this test measuring bradykinesia, the animal was put head‐upward on the top of a vertical pole with a rough surface (height 100 cm; diameter 2.5 cm). The stopwatch was immediately started when the rat was put on the pole. The time to turn and total time to descend from the pole were recorded in three trials for each rat with a maximum time limitation of 120 s. The average of three trials was considered the final score (Matsuura et al. 1997).

#### 2.7.4 Rotarod Test

The rotarod test is one of the most common assays for motor coordination and balance assessment in rats (Buccafusco 2009). This test was assessed using a rotarod apparatus as described previously (Iravanpour et al. 2021). The speed of the rotarod on the first day of training was constant at 10 rpm, and it was accelerating from 5 to 20 rpm during 300 s on the second training day. On testing days, the speed of the rotarod was increased at a fixed rate from 5 to 40 rpm over 300 s. The latency to fall of each animal was recorded with a cutoff of 300 s. The average of 3 trials for each rat was considered the final score.

#### 2.7.5 Apomorphine‐Induced Rotations Test

Apomorphine is a nonselective DA receptor agonist that causes contralateral rotations to the lesion in low doses as a motor dysfunction index by stimulation of supersensitive D1 and D2 receptors, mostly in the denervated side (Calou et al. 2014; Ünal et al. 2020). Apomorphine (0.25 mg/kg, dissolved in 0.02% ascorbate in normal saline) subcutaneous injections were done on testing days to evaluate the turning behavior of each rat (Miyanishi et al. 2019). Then, after 5 min, the net contralateral rotations were calculated for 30 min.

### 2.8 Expression and Purification of α‐Syn

α‐syn expression and purification followed the method outlined in the study by Marvastizadeh et al. (2020). α‐syn was expressed in *E. coli* BL21. Cultivation of cells occurred in LB medium supplemented with kanamycin (50 μg/mL) at 37°C and 180 rpm. After attaining an absorbance of 0.5 at 600 nm (OD600), the expression was induced using 1 mM IPTG for 4 h. The cells were harvested, suspended in lysis buffer (50 mM Tris, 5 mM imidazole, 2 M NaCl), and disrupted using sonication. Centrifugation (12,000 g, 20 min at 4°C) was used to remove the disrupted cells, and the samples were placed in a water bath at 100°C to eliminate impurities. To purify the protein, the supernatant was loaded onto a Ni‐agarose column. The column was eluted with a linear gradient from 5 to 60 mM imidazole (pH = 7.8) and to separate the protein from the column, 250 mM imidazole (pH = 7.8) buffer was used. The purity of eluted fractions was verified using SDS‐PAGE electrophoresis (Figure S2).

The dialysis of the purified α‐syn was performed using a 2‐kDa dialysis membrane against 30 mM Tris buffer and 200 mM NaCl (pH = 7.8) three times for 16 h. α‐syn concentration was determined using a molar absorption coefficient of ε = 5.960 cm^−1^ M^−1^. Fibril formation was conducted in a mixture containing α‐syn (2 mg/mL) in 30 mM Tris buffer (pH = 7.8) and 200 mM NaCl at 37°C while stirred using micro stir‐bars. To detect the inhibitory effect of PGHE on α‐syn fibril formation, different concentrations of PGHE were added to a solution of α‐syn (2 mg/mL) and incubated under the mentioned condition.

### 2.9 α‐Syn Fibril Detection

To determine the inhibitory impact of PGHE on α‐syn fibrillation, the following tests were carried out.

#### 2.9.1 Thioflavin T Fluorescence Assay

At different time intervals, 10 μL of incubated protein was withdrawn and mixed with 990 μL of 25 μM thioflavin T (ThT, in 25 mM phosphate buffer, pH = 6.0), and then, in the presence and absence of PGHE, the fibrillation of the protein was investigated. Subsequently, the sample was excited at 440 nm, and the resulting ThT fluorescence spectra were recorded from 450 to 600 nm using LS55 Perkin Elmer Spectrofluorometer (Waltham, MA, USA). ThT intensity at 485 nm versus time was plotted. Each experiment was performed three times (Honarmand et al. 2019).

#### 2.9.2 Congo Red Assay

Congo red solution must be freshly prepared. The stock solution supplied was a 50x concentration. Therefore, 7 mg of powder was dissolved in 5 mM potassium phosphate containing 150 mM NaCl. Subsequently, the solution was filtered using a 0.2 μm syringe filter and then diluted 50‐fold. After that, 440 μL of the diluted solution was added to 60 μL of the protein solutions. The spectra of samples in the presence and absence of PGHE were recorded from 400 to 700 nm using a Perkin Elmer UV–Vis spectrophotometer (Waltham, MA, USA). Each experiment was performed three times (Marvastizadeh et al. 2020).

#### 2.9.3 Transmission Electron Microscopy (TEM)

For studying α‐syn aggregates, 20 μL of incubated protein in the presence and absence of PGHE were placed on carbon grids. Then, they were stained with uranyl acetate (2%), and TEM images were observed using TEM Philips EM 208S 100 kv transmission electron microscopy after 5 min (Honarmand et al. 2019).

### 2.10 Statistical Analysis

To analyze the data from all behavioral tests (in vivo tests), GraphPad Prism 9.5.1 software was used (GraphPad Software, San Diego, CA, USA). Results were expressed as mean ± SEM, and the significance level was *p* < 0.05. The normal distribution was determined by the Kolmogorov–Smirnov test. The data statistical analysis was done by one‐way ANOVA followed by Tukey's post hoc test between groups for each testing day. Two‐way ANOVA followed by Tukey's post hoc test for evaluation of the treatment duration effects was also carried out. All in vitro experiments were performed in triplicate, and the results were reported as mean ± standard deviation. To analyze the mean values, one‐way ANOVA followed by Tukey's HSD post hoc test was used. A *p*‐value of 0.05 was considered statistically significant. To determine the differences between the means, SPSS v. 24 was used.

## 3 Results and Discussion

### 3.1 Identification of PGHE Phenolic Compounds

**FIGURE 1:**
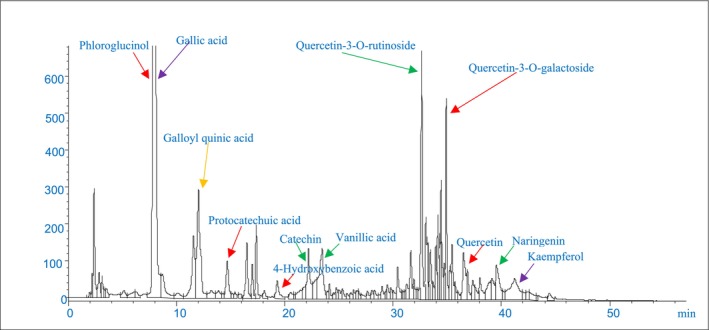
The LC/MS chromatogram of PGHE phenolic compounds.

As depicted in the LC/MS chromatogram (Figure 1), gallic acid, phloroglucinol, quercetin‐3‐O‐rutinoside, and quercetin‐3‐O‐galactoside were the most abundant phenolic compounds in PGH aqueous extract, consistent with prior studies (Barreca et al. 2016; Lalegani et al. 2018).

### 3.2 Acute Oral Toxicity of PGHE

The acute toxicity study determines the lethal dose (LD_50_) of the pharmacological agents. LD_50_ means the dose of a substance that causes 50% mortality in test animals (Chinedu et al. 2013). In this research, the acute toxicity study of PGHE showed no mortality in the studied groups up to a single administered dose of 5000 mg/kg after 24 h and 14 days. Furthermore, the animals gained weight over 14 days of the study and appeared healthy and active (data not shown). Based on this condition of acute oral toxicity study, the LD_50_ of PGHE was greater than 5000 mg/kg, and therefore PGHE has a high degree of safety and is practically nontoxic (Chinedu et al. 2013; Hayes et al. 2020).

### 3.3 Behavioral Tests

In the 6‐OHDA‐induced model of PD, the unilateral denervation induces some behavioral dysfunctions similar to patients with Parkinson's due to a fall in dopamine. So, various behavioral tests are expanded in PD models to assess the lesion intensity and treatment effects (Calou et al. 2014; Monville et al. 2006). In this study, the behavioral evaluations were performed on Days 7 and 15 after surgery.

#### 3.3.1 Cylinder Test

**FIGURE 2:**
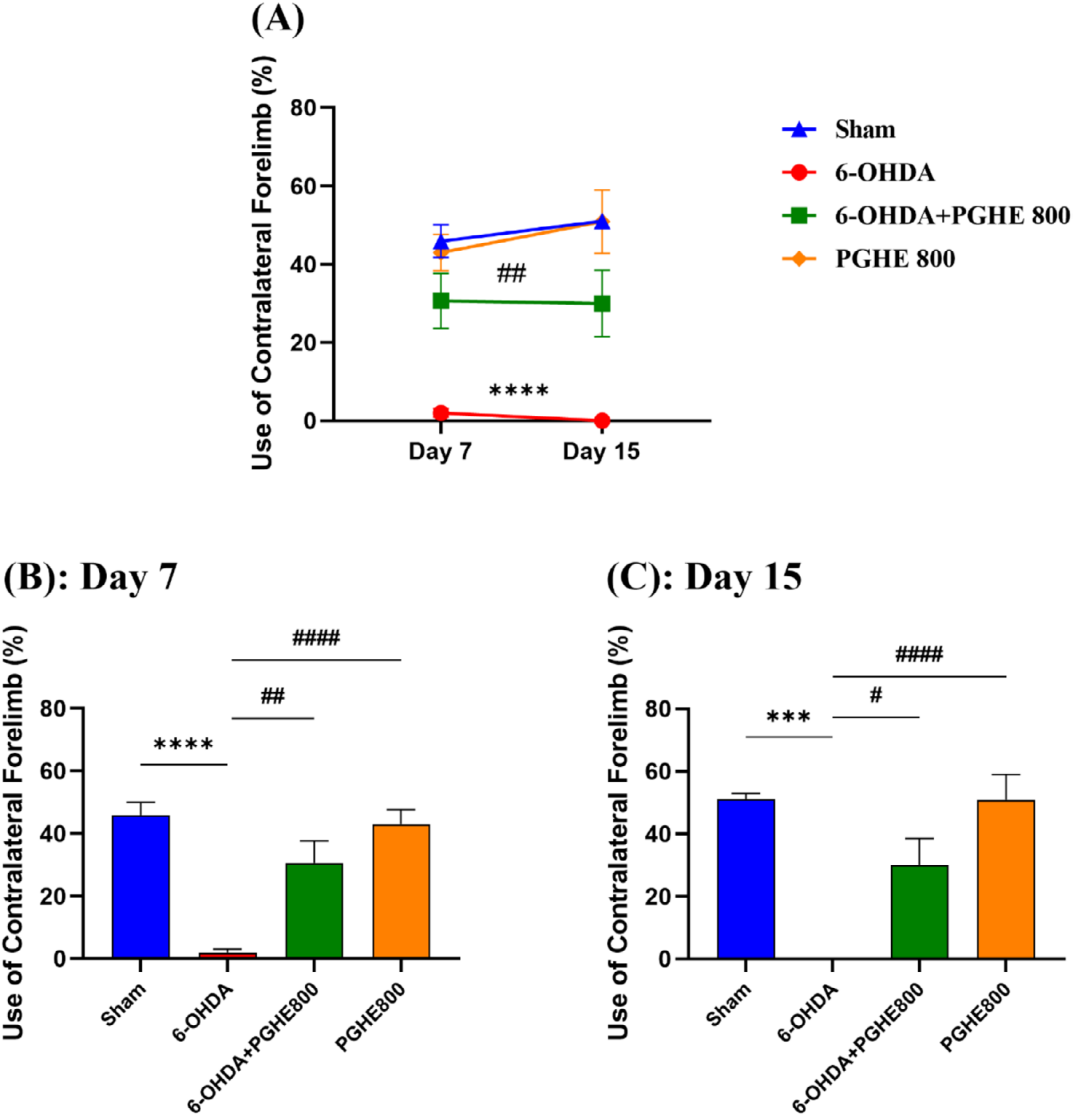
Effect of 15 days of treatment with PGHE on the use of contralateral forelimb. ***p < 0.001, and ****p < 0.0001 compared to Sham and #*p* < 0.05, #*#p* < 0.01, and #*###p* < 0.0001 compared to 6‐OHDA using repeated measures (A) and one‐way ANOVA (B, C) followed by Tukey's multiple comparisons. All values are expressed as mean ± SEM (*n* = 7–9/group).

In this study, the left limb was defective (contralateral to the lesion); therefore, the contralateral paw use percentage was calculated. Repeated measures (Figure 2A) showed a significant effect of treatments [*F*_(3,27)_ = 20.07, *p* = 0.0001] on the use of the contralateral forelimb. Tukey's post hoc analysis showed significant differences between the 6‐OHDA and treatment group (6‐OHDA+PGHE 800), as well as the sham group. In the cylinder test, rats with unilateral lesions used their contralateral paw less than the sham rats. Therefore, the cylinder test can measure the degree of unilateral dopamine loss (Hemmati et al. 2023; Soner et al. 2021). Therefore, these differences indicated the successful induction of disease by decreased use of the contralateral forelimb in the 6‐OHDA group compared with the sham group (*p* < 0.0001); and PGHE treatment improved the use of the contralateral forelimb in 6‐OHDA+PGHE800 group compared with 6‐OHDA group (*p* = 0.0011). Moreover, repeated measures did not show significant effects of time [*F*_(1,25)_ = 0.4428, *p* = 0.5119] and time × treatment interaction [*F*_(3,25)_ = 0.5072, *p* = 0.6809] on the use of contralateral forelimb. One‐way ANOVA showed the successful induction of disease (Sham vs. 6‐OHDA) and the positive effect of PGHE treatment (6‐OHDA vs. 6‐OHDA+PGHE) on the use of contralateral forelimb percentage on days 7 (Figure 2B) and 15 (Figure 2C). So, these findings showed that PGHE treatment affects the attenuation of Parkinson's disease motor impairments by decreasing unilateral dopamine loss. A previous study showed that pre‐ and post‐pistachio treatment (800 mg/kg/day, p.o. for 2 weeks) exerted neuroprotective effects by improving motor deficits, increasing DA levels, and attenuating oxidative stress in a rotenone‐induced Parkinson's disease rat model (Haider et al. 2020). Moreover, as shown in Figure 2, PGHE 800 had almost equal performance compared to the sham group, indicating that PGHE did not have negative effects on animals.

#### 3.3.2 Narrow Beam Test

**FIGURE 3:**
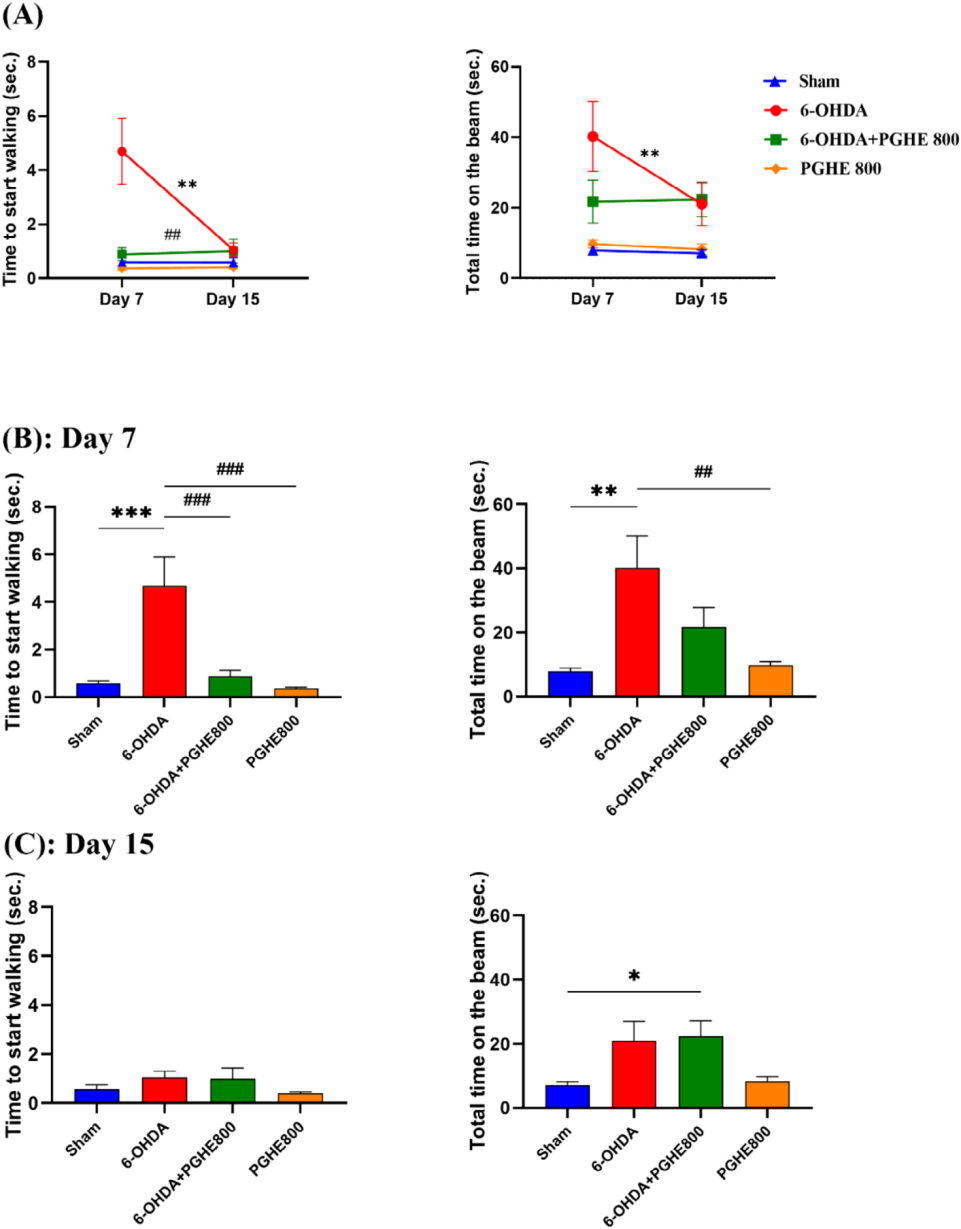
Effect of 15 days of treatment with PGHE on motor performance and coordination using narrow beam test. The time to start walking and total time on the beam were measured 7 and 15 days after the 6‐OHDA injection. *p < 0.05, **p < 0.01, ***p < 0.001 compared to Sham and ##*p* < 0.01, ###*p* < 0.001 compared to 6‐OHDA using repeated measure (A) and one‐way ANOVA (B, C) followed by Tukey's multiple comparisons. All values are expressed as mean ± SEM (*n* = 8–9/group).

This task examines the animal's ability to walk on an elevated narrow beam. So, this test can detect the loss of nigrostriatal dopamine (Buccafusco 2009). As shown in Figure 3A, repeated measures yielded a significant time effect [*F*_(1,29)_ = 8.665, *p* = 0.0063], reflecting the increased activity during 15 days of evaluations, treatment effect [*F*_(3,32)_ = 8.085, *p* = 0.0004], indicating overall differences between the groups, and time × treatment interaction [*F*_(3,29)_ = 9.323, *p* = 0.0002], indicating significant differences between the groups over time on time to start walking. Tukey's post hoc analysis indicated significant differences between 6‐OHDA and the treatment group (6‐OHDA + PGHE 800) as well as the sham. These differences indicate the successful induction of disease by increases in time to start walking (6‐OHDA vs. sham) and the positive effect of PGHE on the performance of animals by decreases in time to start walking (6‐OHDA + PGHE800 vs. 6‐OHDA). Also, analysis of total time on the beam showed a significant effect of treatment (6‐OHDA vs. sham), indicating induction of disease (*p* = 0.0036) within 7 days (Figure 3A,B). Therefore, these observations confirm the effective oral treatment of PGHE on significant degeneration due to 6‐OHDA injection. Bradykinesia as a marker of motor impairment measured by this test improved through PGHE treatment. As shown in Figure 3A,B, the PGHE oral administration notably decreased the time to start walking compared to 6‐OHDA in rats on Day 7 (*p* < 0.001). A previous study investigating the effects of quercetin found that it reduced the time to cross the narrow beam in rotenone‐ and iron supplement–treated rats (Sharma et al. 2020). Although the difference between treatment and 6‐OHDA groups was not significant on day 7 for total time to cross the beam, the graph shows the effective oral treatment of PGHE in time decline. 6‐OHDA as a catecholaminergic toxin causes an extreme decline of dopaminergic neurons in SNpc. Dopamine depletion in the striatum leads to a delay in time to start walking and total time to cross the beam. These delays indicate akinesia and bradykinesia, balance, and postural instability in animals affected by Parkinson's disease, respectively (Allbutt and Henderson 2007). Motor impairment occurs when 60%–70% of dopaminergic neurons are lost; with less damage, animals can gradually recover the mentioned movement disorders such as akinesia and bradykinesia (Matsuura et al. 1997). So, this recovering behavior on day 15 of the beam test of the 6‐OHDA group (Figure 3A,C), can be due to less damage of the nigrostriatal pathway. The PGHE treatment may improve the rat's performance on the narrow beam by reducing oxidative stress through antioxidant defense system enhancement, and as a result, leading to loss prevention of SNpc dopaminergic neurons. Kesh et al. (2021) reported that naringenin, which is also a component of PGHE, reduces oxidative stress biomarkers induced by 6‐OHDA in SHSY5Y cells. Moreover, PGHE treatment demonstrated no adverse effects on the performance of the examined animals, as the performance of the PGHE800 group was almost equivalent to that of the sham group (Figure 3A,B).

#### 3.3.3 Pole Test

**FIGURE 4:**
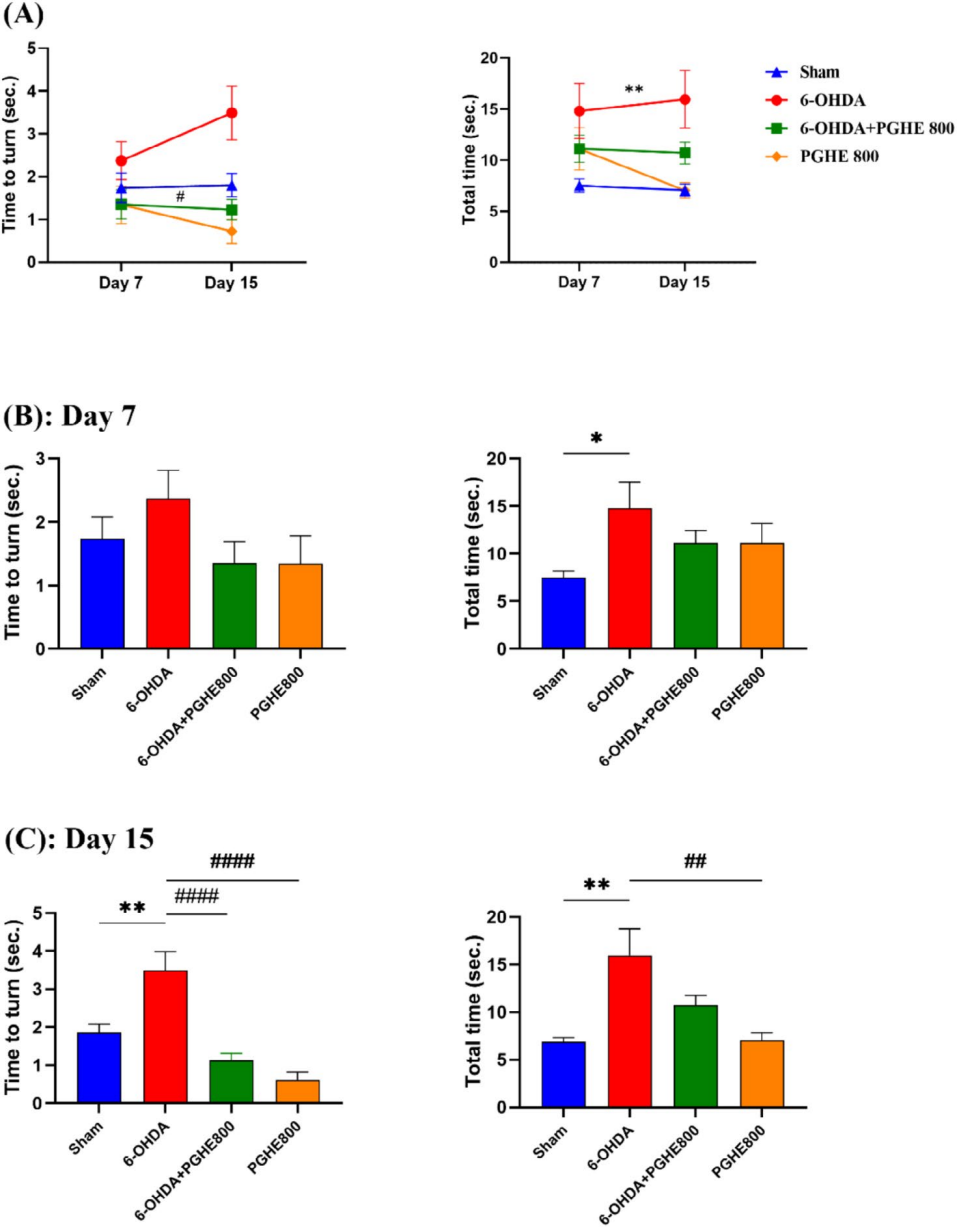
Effect of 15 days treatment with PGHE on bradykinesia using pole test. Time to turn and total time were measured 7 and 15 days after 6‐OHDA injection. *p < 0.05, **p < 0.01 compared to Sham and #*p* < 0.05, ##*p* < 0.01, ###*#p* < 0.0001 compared to 6‐OHDA using repeated measure (A) and one‐way ANOVA (B, C) followed by Tukey's multiple comparisons. All values are expressed as mean ± SEM (*n* = 8–9/group).

This task needs intact basal ganglia and rubrospinal pathway activation for skilled forelimb grasping and maneuvering. Therefore, the pole test is so sensitive to nigrostriatal dysfunction. As shown in Figure 4A, repeated measures did not show a significant effect of time [*F*_(1,27)_ = 0.1598, *p* = 0.6925] and time × treatment interaction [*F*_(3,27)_ = 2.439, *p* = 0.0862] on time to turn. Nonetheless, the treatments had a significant effect [*F*_(3,32)_ = 6.107, *p* = 0.0021] on time to turn. Tukey's post hoc analysis indicated significant differences between 6‐OHDA and the treatment group (6‐OHDA + PGHE 800). These differences indicate the positive effect of PGHE on the performance of animals by time‐to‐turn reduction compared to the 6‐OHDA group. Also, analysis of total time showed a significant difference between the 6‐OHDA and sham group (*p* = 0.0023), indicating induction of disease [*F*_(3,32)_ = 5.673, *p* = 0.0031]. Meanwhile, time and time × treatment interaction didn't have a significant effect. Figure 4B shows the induction of PD by significant differences in total time between sham and 6‐OHDA groups (*p* = 0.0373) on day 7. Moreover, Figure 4C demonstrates the induction of disease by significant differences between sham and 6‐OHDA groups in time to turn and total time. As shown in Figure 4C, the PGHE oral administration notably decreased the time to turn (*p* < 0.0001) compared to 6‐OHDA on day 15. These results confirm the positive effect of PGHE on the bradykinesia of rats. Therefore, to observe the positive effects of PGHE on the performance of animals in the pole test, a longer treatment duration of 15 days is required. Also, PGHE did not have any adverse effect on animals as shown in Figure 4.

#### 3.3.4 Rotarod Test

**FIGURE 5:**
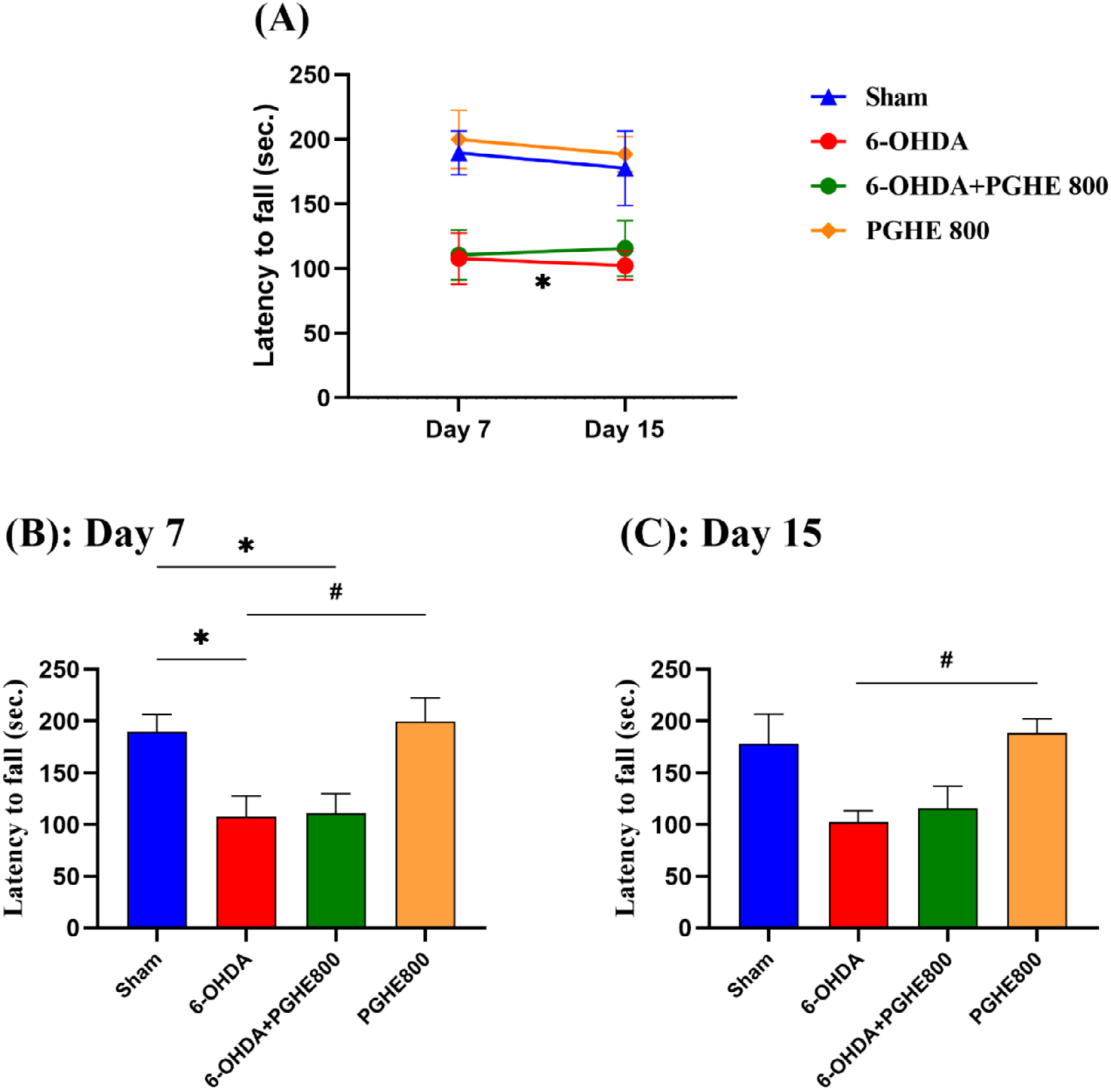
Effect of 15 days treatment with PGHE on motor coordination in rotarod apparatus against rat model of 6‐OHDA‐induced PD. *p < 0.05 compared to Sham and #*p* < 0.05 compared to 6‐OHDA using repeated measures (A) and one‐way ANOVA (B, C) followed by Tukey's multiple comparisons. All values are expressed as mean ± SEM (*n* = 8–9/group).

The rotarod test is used to evaluate motor coordination and balance in rodents. The time spent on rotating bars by animals is inversely related to the loss of neurons (Calou et al. 2014). As shown in Figure 5, the sham‐operated control group showed a stable performance over 15 days of study. Whereas, rats with Parkinson's disease can maintain themselves less on rotarod apparatus compared to the sham group since they cannot keep their motor coordination and balance. A significant treatment effect [*F*_(3,31)_ = 7.136, *p* = 0.0009] indicates overall differences between groups in this test. Repeated measures (Figure 5A) showed the induction of disease in the rat model by decreased latency to fall in the 6‐OHDA group compared to the sham group (*p* = 0.0146). This confirmation of disease induction is also shown in the one‐way ANOVA analysis of day 7 (*p* = 0.0311). 6‐OHDA administration in rats causes a decline in motor coordination. Meanwhile, all graphs of Figure 5 did not show a significant protective effect of PGHE treatment against deterioration caused by 6‐OHDA on the rotarod apparatus. Nevertheless, we can also assess posture and steps in rats with brain injuries using the rotarod test (Monville et al. 2006). Our observations during the experiment showed the improved stepping of the 6‐OHDA + PGHE 800 group compared to the 6‐OHDA group. So, it may be due to the positive effect of PGHE treatment in contrast to statistical analysis of latency to fall. Treatment with the combination of vanillic acid, a compound also present in PGHE, and levodopa carbidopa has been reported to significantly improve muscle coordination in a rotenone‐induced Parkinson's disease rat model (Sharma et al. 2021). Furthermore, PGHE 800 has an equal function compared to the sham group. So, similar to other tests, PGHE 800 has no negative effect on animals alone.

#### 3.3.5 Apomorphine‐Induced Rotations Test

**FIGURE 6:**
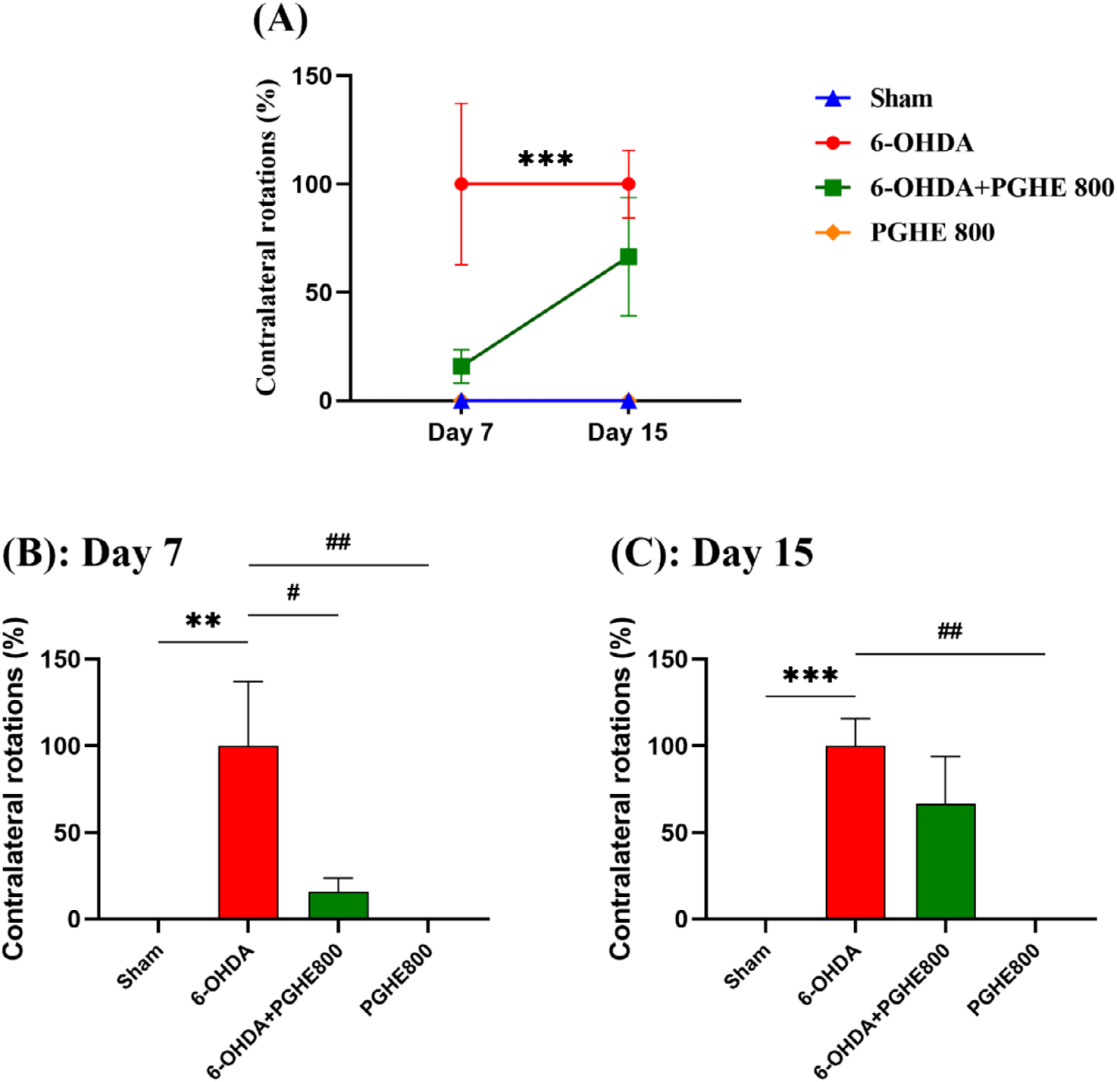
Effect of 15 days treatment with PGHE on apomorphine induced rotational behavior compared to rat model of 6‐OHDA‐induced PD. **p < 0.01, and ***p < 0.001 compared to Sham and #*p* < 0.05, and ##*p* < 0.01 compared to 6‐OHDA using repeated measures (A) and one‐way ANOVA (B, C) followed by Tukey's multiple comparisons. All values are expressed as mean ± SEM (*n* = 8–9/group).

In the rat models, the apomorphine‐induced rotational test is widely used to assess the effects of 6‐OHDA neurotoxin. Whereas the behavior of the lesioned rats is completely normal before apomorphine injection (Hudson et al. 1993). Repeated measures ANOVA of the percentage of the contralateral rotations (Figure 6A) revealed significant effects of treatments [*F*_(3,32)_ = 8.221, *p* = 0.0003] and time [*F*_(1,28)_ = 4.568, *p* = 0.0414]. Whereas the effect of time × treatment interaction was not significantly different. The contralateral rotation number induced by apomorphine injection directly relates to the dopaminergic system damage (Huang et al. 2018). As seen in Figure 6A, significant contralateral rotations (leftward) in the 6‐OHDA lesioned rats compared to the sham group (*p* = 0.0008) indicate the successful induction of PD in the rat model. The minimum time required for induction of PD was 7 days as depicted in Figure 6B,C. Moreover, the PGHE treatment attenuated the contralateral rotation compared to the 6‐OHDA lesioned group (64.53% ± 24.84%) but it is not statistically significant as in Day 15 (*p* = 0.4762; Figure 6C). As shown in Figure 6B, the PGHE treatment significantly reduced the apomorphine‐induced rotational behavior by 84.20% ± 28.51% after 7 days compared to the 6‐OHDA lesioned group (*p* = 0.0293). Based on previous studies on the intense antioxidant feature of PGHE (Goli et al. 2005; Rafiee et al. 2018; Rajaei et al. 2010; Sadeghinejad et al. 2019; Sarteshnizi et al. 2019), these results indicated the positive effect of the PGHE on dopaminergic neurons loss prevention by scavenging free radicals in SNpc; as a result, motor dysfunction decreases. So, these observations indicated the PGHE protective effect against the 6‐OHDA‐induced motor functional impairments in the rat Parkinson's model. As depicted in Figure 6, similar to other behavioral tests, PGHE showed no bad effects on the performance of animals in the apomorphine‐induced rotational test.

### 3.4 Inhibitory Effect of PGHE on Fibrillation of α‐Syn

α‐syn is a normal protein present in all neurons; however, in PD, it undergoes misfolding and aggregates into the toxic fibrillar structure (Negi et al. 2024). Research has shown that preventing or reducing the fibrillation of α‐syn can play an important role in reducing the symptoms of α‐syn‐related diseases such as PD (Bellucci et al. 2012). Focusing on α‐syn offers an effective approach to slowing the progression of PD (Singh et al. 2021).

**FIGURE 7:**
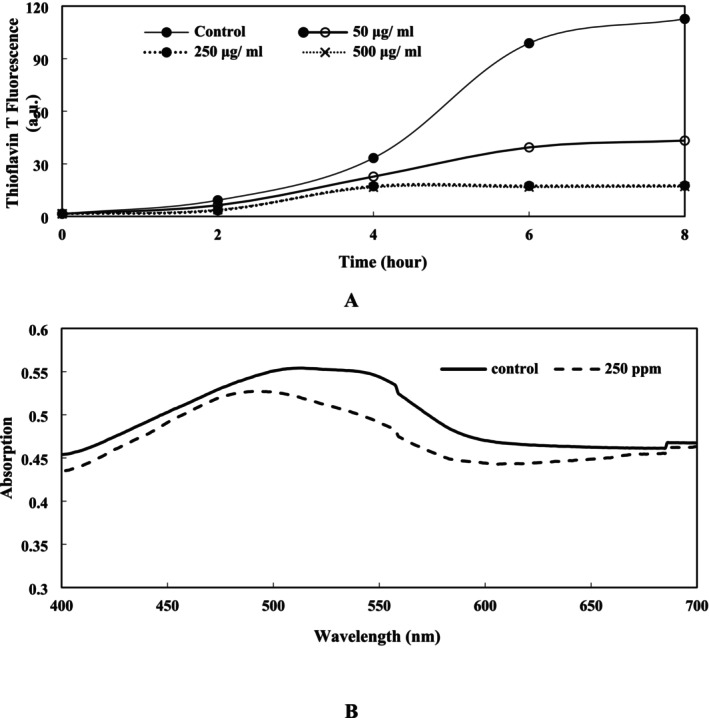
Inhibitory effect of PGHE on α‐synuclein fibril formation. (A) Thioflavin test; (B) Congo red test in the absence and presence of 250 μg/ml of pistachio green hull extract.

The fluorescence of ThT increased in the control and sample containing 50 μg/mL of PGHE (Figure 7A), which indicates an increase in the progress of α‐syn fibrillation. This shows that α‐syn underwent a conformational transition to a cross β‐sheet rich structure leading to the formation of α‐syn fibrils during this specified time. It can be concluded that the highest fibril formation was observed in the control group. The ThT assay revealed that 250 and 500 μg/mL of extract have higher inhibitory effects (84%) than samples containing 50 μg/mL of PGHE (60%). Therefore, 250 μg/mL of extract was chosen as the optimum concentration. Polyphenols have been reported to inhibit the formation of fibrils by preserving the native conformation of α‐syn and preventing the development of β‐sheet structures, which are critical for amyloid fibril formation. In addition, various flavonoids such as Baicalein have been shown to inhibit α‐synuclein fibrillation (Meng et al. 2010). In this context, the high concentration of bioactive compounds, including polyphenols, in PGHE has been previously confirmed (Arjeh et al. 2020), further supporting its potential role in reducing amyloid fibrillation.

Congo red assay was also applied to study α‐syn fibrillation. A commonly used histological dye for α‐syn detection is Congo red. The specificity of this staining arises from Congo red's affinity for binding to fibril proteins enriched in β‐sheet conformation (Frid et al. 2007). The interaction between Congo red and the fibrils rich in β‐sheet structure is accompanied by an increase in absorption and a red shift from 480 to 540 nm (Figure 7B). Changes in the absorption spectra of Congo red in the wavelength range of 400–600 nm for the control and 250 μg/mL of the extract were investigated after 8 h of incubation. The enhancement in the absorption spectra and a red shift in the absorption maximum of the control sample indicates the presence of more fibrillar structures and the inhibition of α‐syn fibrillation at 250 μg/mL of the extract.

### 3.5 Transmission Electron Microscopy (TEM)

**FIGURE 8:**
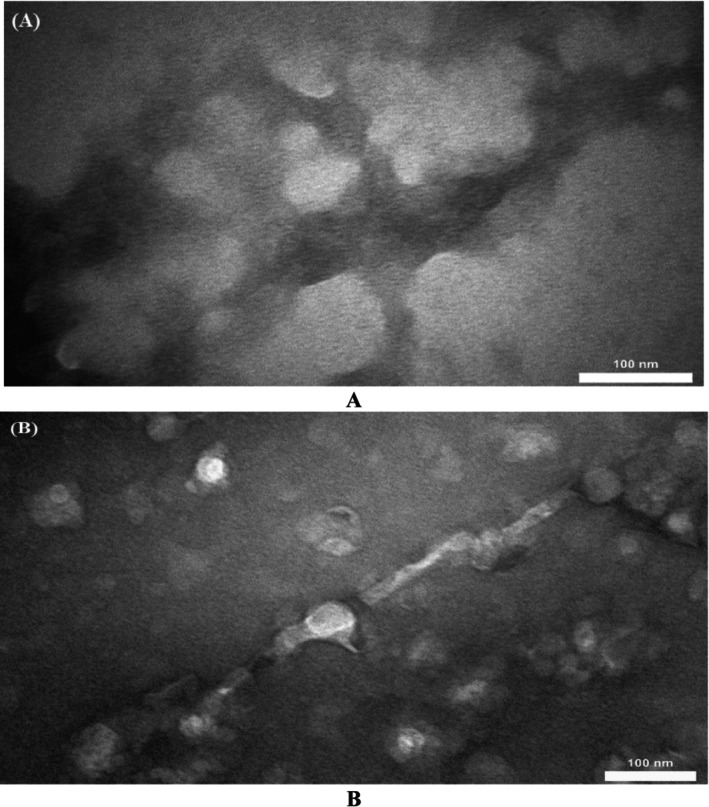
TEM images captured from α‐syn after 8 h of incubation at 37**°**C. (A) In the absence and (B) in the presence of 250 μg/ml of PGHE. Scale bar = 100 nm.

TEM was employed to confirm the findings from the ThT and Congo red assay and to visually demonstrate the fibril formations in the presence and absence of PGHE (Figure 8). As shown in Figure 8A, rope‐like mature α‐syn fibrils were observed when α‐syn was incubated alone. However, the large amount of α‐syn fibrils has noticeably diminished, with a minimal amount of small oligomers observed in the presence of extract (Figure 8B).

## 4 Conclusions

In this study, anti‐Parkinson's disease properties of PGHE were evaluated in vivo and in vitro. The acute oral toxicity study found PGHE safe and practically nontoxic (LD_50_ > 5000 mg/kg). Eight hundred mg/kg BW of PGHE showed significant positive effects on behavioral performance in the rat model of PD by improvements in performing cylinder, narrow beam, pole, and apomorphine‐induced rotational tests. Based on these results, it is assumed that PGHE can attenuate the oxidative stress of dopaminergic neurons. Moreover, an in vitro study showed that 250 μg/ml of PGHE had the highest inhibitory effect on the formation of α‐syn fibril. Therefore, PGHE can be considered an inhibitor of α‐syn fibrillation. These findings show the potential anti‐PD activity of PGHE. Therefore, PGHE can be considered a novel supplement for PD treatment. Assessing the levels of α‐syn aggregation and related biochemical changes in the brain is recommended for future studies to comprehensively evaluate the effects of PGHE.

## Author Contributions

**Maedeh Tavakoli:** data curation (equal), formal analysis (equal), investigation (equal), writing – original draft (equal). **Negin Mohammadi:** data curation (equal), formal analysis (equal), investigation (equal), writing – original draft (equal). **Mohsen Barzegar:** conceptualization (equal), funding acquisition (equal), project administration (equal), supervision (equal), writing – review and editing (equal). **Neda Valian:** data curation (equal), investigation (equal), methodology (equal), software (equal), writing – review and editing (equal). **Narges Hosseinmardi:** data curation (equal), investigation (equal), methodology (equal), writing – review and editing (equal). **Mohammad Ali Sahari:** investigation (equal), writing – review and editing (equal). **Sabereh Saremi:** investigation (equal), methodology (equal).

## Ethics Statement

The research Ethics Committees of Tarbiat Modares University approved the care and use of laboratory animals (IR.MODARES.REC.1401.019).

## Conflicts of Interest

The authors declare no conflicts of interest.

## Supporting information


**Figure S1.** The chart of experimental design.

**Figure S2.** The SDS‐PAGE electrophoresis of eluted fractions (lanes 1 and 2: 30 mM imidazole; lane 3: 45 mM imidazole; lanes 4 and 5: 60 mM imidazole; lanes 6–9: 250 mM imidazole). α‐syn has a molecular mass of 14 kDa but it migrates on SDS‐PAGE with the apparent mass of ~20 kDa due to its poor binding to SDS.

## Data Availability

Data will be made available on request.
