# Supplementary figures and images for: Therapeutic Potential of Pistachio Green Hull Extract in Treating Parkinson's Disease: A Comprehensive In Vivo and In Vitro Investigation
Source: Food Sci Nutr. 2025 May 16;13(5):e70204. doi: 10.1002/fsn3.70204 (PMC12082081; doi:10.1002/fsn3.70204)

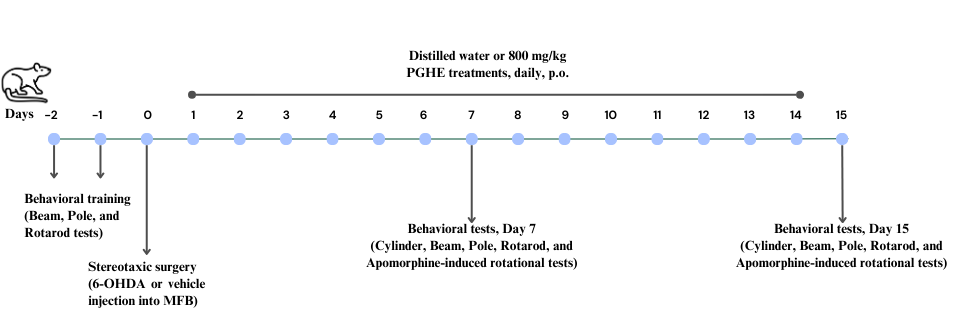


**Figure S1.** The chart of experimental design.

Supplement: Supplementary file 1 — Figure S1. The chart of experimental design. [file FSN3-13-e70204-s001.docx]
