# Supplementary material for: Therapeutic Potential of Pistachio Green Hull Extract in Treating Parkinson's Disease: A Comprehensive In Vivo and In Vitro Investigation
Source: Food Sci Nutr. 2025 May 16;13(5):e70204. doi: 10.1002/fsn3.70204 (PMC12082081; doi:10.1002/fsn3.70204)
Supplement: Supplementary file 2 — Figure S2. The SDS‐PAGE electrophoresis of eluted fractions (lanes 1 and 2: 30 mM imidazole; lane 3: 45 mM imidazole; lanes 4 and 5: 60 mM imidazole; lanes 6–9: 250 mM imidazole). α‐syn has a molecular mass of 14 kDa but it migrates on SDS‐PAGE with the apparent mass of ~20 kDa due to its poor binding to SDS. [file FSN3-13-e70204-s002.docx]

**
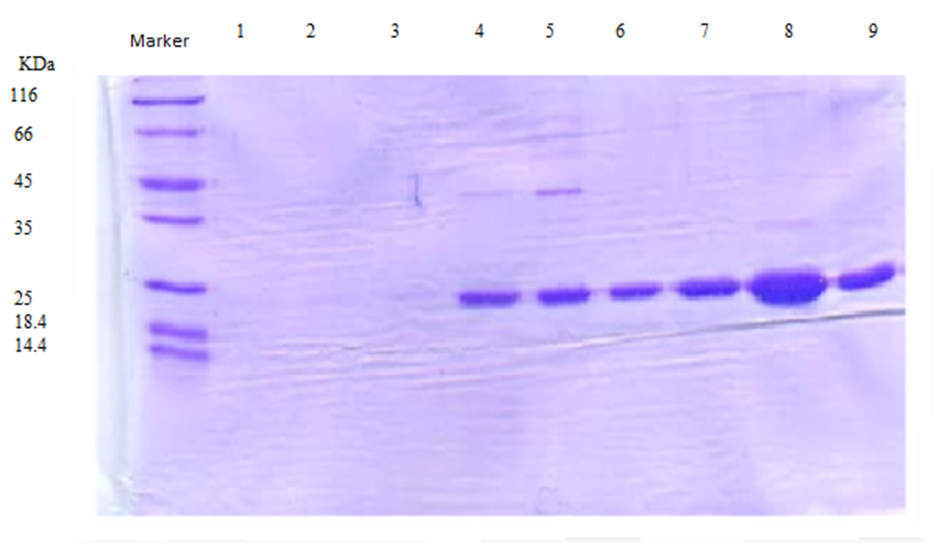
**

**Figure S2.** The SDS-PAGE electrophoresis of eluted fractions (lanes 1 and 2: 30 mM imidazole; lane 3: 45 mM imidazole; lanes 4 and 5: 60 mM imidazole; lanes 6 to 9: 250 mM imidazole. α-synuclein has a molecular mass of 14 kDa but it migrates on SDS-PAGE with the apparent mass of ~ 20 kDa due to its poor binding to SDS.
